# Efficacy of virtual reality training on motor performance, activity of daily living, and quality of life in patients with Parkinson's disease: an umbrella review comprising meta-analyses of randomized controlled trials

**DOI:** 10.1186/s12984-023-01256-y

**Published:** 2023-09-30

**Authors:** Jingxuan Yu, Jinlong Wu, Jiancong Lu, Xijun Wei, Kangyong Zheng, Bowen Liu, Wen Xiao, Qiuqiong Shi, Lilin Xiong, Zhanbing Ren

**Affiliations:** 1 College of Physical Education, Shenzhen University, Shenzhen, 518060 China; 2 College of Physical Education, Southwest University, Chongqing, 400000 China; 3 Department of Neurology, Foshan First People’s Hospital, Foshan, 528000 China; 4 Department of Rehabilitation Medicine, Shenzhen Hospital of Southern Medical University, Shenzhen, 518101 China; 5 Department of Rehabilitation Sciences, The Hong Kong Polytechnic University, Hong Kong, 999077 China; 6 Laboratory for Artificial Intelligence in Design, Hong Kong, 999077 China; 7 College of Physical Education, Kookmin University, Seoul, 02707 South Korea

**Keywords:** Parkinson's disease, Virtual reality training, Activity of daily living, Quality of life, Motor performance

## Abstract

**Objective:**

There are several meta-analyses of randomized controlled trials (RCTs) demonstrating the benefits of virtual reality (VR) training as an intervention for motor performance, activity of daily living (ADL) and quality of life (QoL) outcomes in patients with Parkinson's disease (PD). However, the aggregate evidence collected to date has not been thoroughly evaluated for strength, quality, and reproducibility. An umbrella review from published meta-analyses of RCTs was conducted to evaluate the strength and quality of existing evidence regarding the efficacy of VR training in improving the motor performance, ADL and QoL outcomes of patients with PD.

**Methods:**

PubMed, PsychInfo, Web of Science, and Scopus were searched to identify relevant meta-analysis of RCTs examining the effects of VR training on motor performance and quality of life outcomes in PD patients. We recalculated the effect sizes (Hedges’g) for VR training using DerSimonian and Laird (DL) random effects models. We further assessed between-study heterogeneity, prediction interval (PI), publication bias, small-size studies, and whether the results of the observed positive studies were better than would be expected by chance. Based on these calculations, the quality of evidence for each outcome was assessed by using the Grading of Recommendations, Assessment, Development, and Evaluations (GRADE) criteria.

**Results:**

Four meta-analysis with eight outcomes included in the umbrella review was recalculated effect size. Pooled results found VR training can large improve the basic balance ability, moderate improve the overall balance capacity and moderate improve the stride length in PD patients. For ADL and QoL, the effect sizes were pooled that suggested VR training can moderate improve ADL and QoL for PD patients. However, no statistically clear evidence was found in walking speed, motor function and gait function during VR training. The analyzed meta-analyses showed low-to-moderate methodological quality (AMSTAR2) as well as presented evidence of moderate-to-very low quality (GRADE). Tow adverse reactions were reported in the included meta-analyses.

**Conclusions:**

In this umbrella review, a beneficial correlation between VR and balance ability, stride length, ADL and QoL in PD patients was discovered, especially for the very positive effect of VR on balance because of two of the eight outcomes related to balance ability showed large effect size. The observations were accompanied by moderate- to very low-quality rating evidence, supporting VR training as a practical approach to rehabilitation.

**Supplementary Information:**

The online version contains supplementary material available at 10.1186/s12984-023-01256-y.

## Introduction

Parkinson's disease (PD) is a complex, persistent, neurodegenerative disorder with a high prevalence between the ages of 60 and 90 that increases with age [1]. The neuropathologic cause of PD arises from the loss of dopaminergic neurons in Lewy bodies and substantia nigra containing alpha-synuclein [2]. Parkinson's dyskinesia is clinically characterized by bradykinesia, tremor, rigidity, and postural instability, when these symptoms are present, PD patients tend to fall [3]. There are no clinically available medications that can have a palliative effect on PD [4], but meta-analyses have shown that most types of physical activity can have an effective effect on gait and quality of life in Parkinson's patients [5, 6]. However, in actual rehabilitation training, it is more difficult to carry out long-term physical training because long-term repetitive physical exercise reduces PD's motivation to participate in rehabilitation training, and because long-term training requires a high level of PD's economic status, safety, and training grounds.

The use of virtual reality (VR) training in rehabilitation has gained attention in recent years. VR is a computer technology that provides users with interactive, immersive, multi-sensory environment [7]. It promotes willingness in individuals to participate in rehabilitation by building a virtual environment and designing many activities in flexible scenarios under the condition of satisfying hearing, vision, and sensation, thus increasing enjoyment of the process [8–11]. VR training improved the physical, psychological, and social health outcomes as well as efficacy and adherence during rehabilitation of individuals with PD [12]. Compared with traditional rehabilitation interventions, VR training can give users a more convenient way to exercise and to make rehabilitation fun. It also offers timely feedback after exercise and responds to motor learning and neurologic plasticity [13].

Systematic review and meta-analyses of multiple published randomized controlled trials (RCTs) have demonstrated that VR training can improve motor performance, ADL and QoL in PD by increasing interest in participation compared to traditional sports interventions. Many meta-analyses have focused on specific health outcomes, yet different measures produce different results. To date, the strength and quality of this evidence has not been synthesized. The aim of this umbrella review is to systematically identify relevant meta-analyses of the effectiveness of VR training on motor performance, ADL and QoL in PD, to summarize the results of existing studies, and to assess the strength of the evidence, providing an overall picture of the benefits of VR training on each motor performance and quality of life-related outcome in PD.

## Methods

This umbrella review followed the Preferred Reporting Items for Systematic Reviews and Meta-Analyses (PRISMA) statement for the improved reporting of systematic reviews [14]. The review protocol was registered with the International Prospective Register of Systematic Reviews (PROSPERO) with the number CRD42023398519.

### Search strategy and eligibility criteria

PubMed, Web of Science, Scopus, and PsycINFO databases from the time of database creation until to August 3, 2023 were each searched to identify meta-analyses of RCTs related to the effect of VR training on motor performance, ADL and QoL outcomes in individuals with PD (Additional file 1: Table S1). No language restriction was applied. Our inclusion and exclusion criteria were based on PICOS (P = participants, I = intervention, C = control, O = outcome, S = study design), as shown in Table 1. We imported records that met the inclusion criteria into Endnote Document Manager20 and removed duplicates. Two reviewers (JL and JW) conducted a second literature search for the title, abstract relevance, and selection of included records. A full-text search of all manuscripts was undertaken to confirm study eligibility. Disputes over inclusion and exclusion of literature were decided by a third reviewer (KZ). Only those quantitative meta-analyses that provided effect sizes and a 95% confidence interval (CI) were included. Meta-analyses where the results were less than two RCTs were excluded. According to the literature inclusion principle of the umbrella review, when the RCTs included in two meta-analyses overlapped, the meta-analysis with the most significant number of RCTs for analysis was chosen [15].

**Table 1:** Selection criteria for meta-analysis

Category	Inclusion criteria	Exclusion criteria
Participants	Individuals with PD	Individuals with other chronic diseases
Intervention	VR training (wearing VR equipment to enter an interactive virtual reality scene, generating a three-dimensional VR environment through computer simulation, providing participants with visual, auditory, haptic, and other simulations, thus enabling active participation in the game), including a variety of modes	Intervention without VR training
Control	Usual care or usual exercise	Other rehabilitation interventions such as aerobics, yoga, stretching, medication and so on
Outcome	Direct measures of motor function such as the Berg Balance Scale, Timed Up and Go test, Unified Parkinson's Disease Rating Scale, walking speed, stride length, and Functional Gait Assessment and quality of life (e.g., 39-item Parkinson's Disease Questionnaire and Activities of Daily Living Questionnaire)	
Study design	Only meta-analysis of RCTs	No meta-analysis or RCTs

*PD*, Parkinson's disease, *RCTs* randomized controlled trials, *VR* virtual reality

### Data extraction

Two reviewers (JL and XW) performed independent information extraction for each included meta-analysis, and when there was a dispute over the extraction of information, we referred the decision to a third reviewer (ZR). The information extracted included the first author, year of publication, outcome, type of VR training, population, duration of experiments, and total participants. The data extracted from each outcome study included the number of individuals in the experimental group, number of individuals in the control group, experimental group means, control group means, observed group standard deviation (SD), and control group SD for quadratic calculation.

### Methodological quality and evidence quality

The quality assessment of the meta-analysis was independently performed using A Measurement Tool to Assess Systematic Reviews (AMSTAR-2) by two reviewers (BL and WX), and in the event of a dispute between two reviewers, the decision will be referred to a third reviewer (JL), as shown in Additional file 1: Table S4 [16].

The quality of evidence per outcome provided in a meta-analysis was assessed by using the GRADE criteria (Additional file 1: Table S5, S6), which allows for a comprehensive assessment of five dimensions including (1) risk of bias in the individual studies, (2) inconsistency, (3) indirectness, (4) imprecision, and (5) publication bias [17]. The strength of evidence was graded as high, moderate, low, or very low using GRADE assessment. GRADE assessment are done independently by two reviewers (QS and LX).

### Data analysis

For each outcome between VR and PD’s motor performance, ADL and QoL, we extracted the mean, sample size (n), and standard deviation (SD) of the individual studies included in each meta-analysis based on the study design and performed RCT repeated meta-analyses to calculate the effect sizes (Hedges' g), the 95% confidence interval (CI) and 95% predictive interval (PI). For the calculation of effect sizes for each meta-analysis, we used the DerSimonian and Laird (DL) random-effects model [18]. The magnitude of the Hedges’ values was interpreted as small (< 0.2), moderate (0.5), or large (> 0.8) effect sizes. The PI explains the heterogeneity across studies and describes the uncertainty of the expected effects that may arise in a new study of the same research question [18]. Heterogeneity was assessed with I^2^ metric [19]. Egger's test refers to using linear regression based on the natural logarithmic value of the wallpaper ratio to measure the pairwise composition of the funnel plot. It is used mainly to assess small-study effect bias. This study can consider a small-study effect when the *P* value is less than 0.05 [20].

Since the true Hedges' g of each meta-analysis is unclear, we used the largest study in each outcome as a reasonable outcome. We performed an excess significance test for each of the included outcomes by testing whether the number of each statistically different outcome (*P* < 0.05) was the same as the expected number of studies, which was the sum of each study at the time of the meta-analysis [21].

Sensitivity analysis for the inclusion of different structures to determine the robustness of each finding with relevance was performed. First, sensitivity analyses by excluding small-size studies (< 25%) from each outcome was done [22]. Then, sensitivity analyses by the Hartung-Knapp-Sidik-Jonkam (HKSJ) method for outcomes with fewer than five RCTs for each meta-analysis according to a random-effects meta-analysis was completed [23]. Statistical analyses were conducted using R version 4.1.3.

## Results

### Study identification

**Fig. 1:**
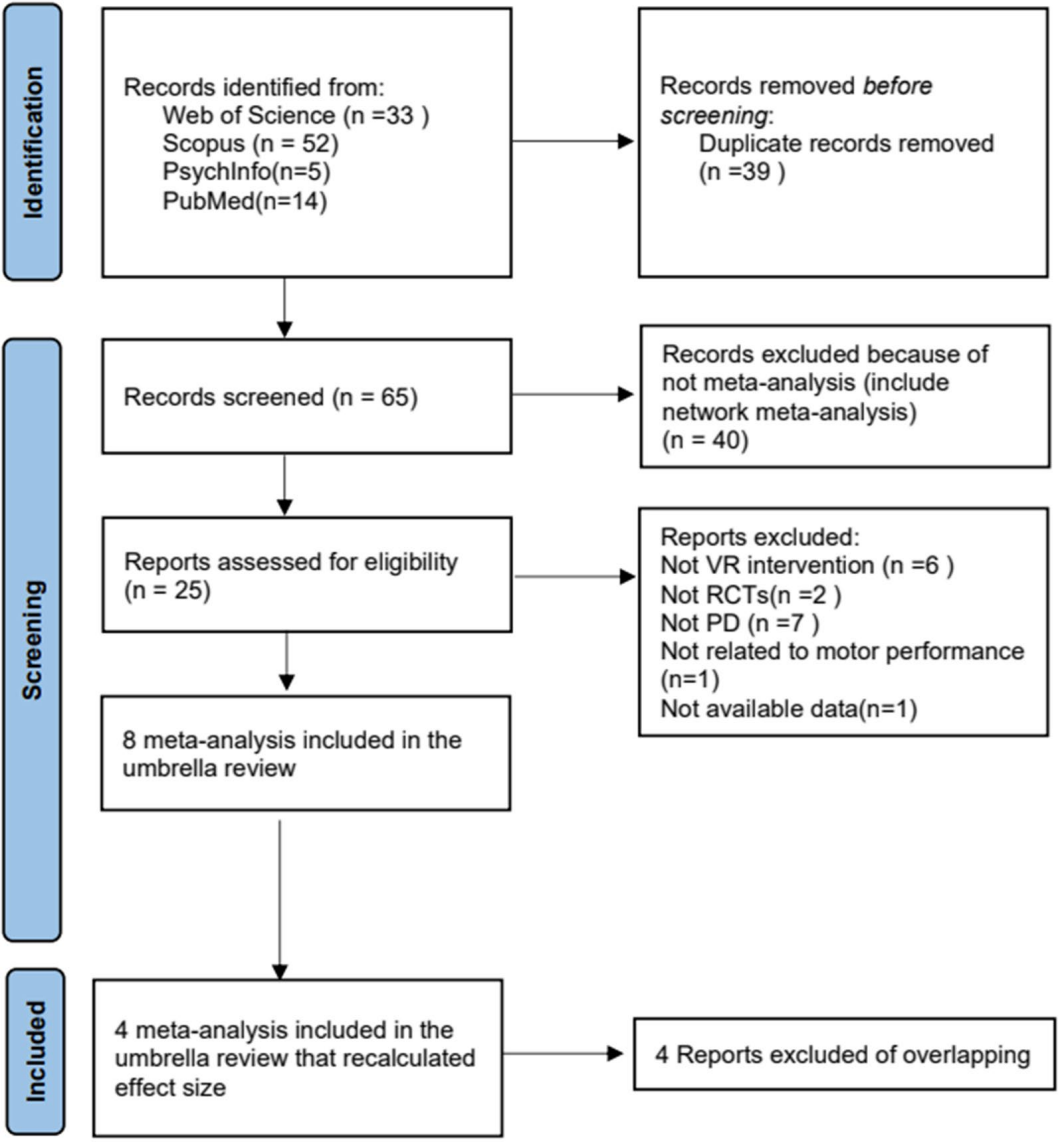
Flowchart of literature included

Based on the literature search strategy, 104 articles were recovered. When duplicates were removed 65 remained. Among these, twenty-five articles met the preliminary data extraction criteria. After applying the literature overlap meta-analysis screening criteria (Fig. 1), eight meta-analysis [11, 24–30] included in the umbrella review and four meta-analysis [11, 27, 29, 30] included in the umbrella review that recalculated effect size. The reasons for the inclusion or exclusion of a study are given in Additional file 1: Table S3.

### Characteristics and quality of each meta-analysis

Eight meta-analyses were included in the umbrella review. The median number of participants was 622 (range 343 to 901) Eight meta-analysis including the 39 unique outcomes on the effects of VR training on motor performance, ADL and QoL. The intervention durations of the included meta-analyses were essentially 3 weeks to 12 weeks. Data on effect were reported in 39 outcomes, of which 28 correlations reported statistically significant results (*P* < 0.05) between the VR training and outcomes. Significant results included the 15 balance correlations (TUG [25–27, 29], BBS [11, 24–30],OLS [28], LOS [28], SOT [28]); 6 gait correlations (stride length [11, 25], 10MWT [26], 6MWT [29], FGA [24] and DGI [24]); 3 QoL correlations (PDQ-39 [11, 28, 30]); 2 ADL correlations (ADL scale [30] and MBI [26]); and 2 feeling correlations (ABC [28] and FES [28]) (Table3). Besides, 11 outcomes reported non statistically significant results (*P* > 0.05), including 3 balance outcomes (ABC [24],TUG [24, 28]), 7 gait outcomes (walking speed [11, 27, 29], gait velocity [25], FGA [29], stride length [29], walk distance [25]) and one motor function outcome (UPDRS [11]).Two studies reported adverse effects, one reported [24] adverse effects associated with VR training and the other reported [27] adverse effects that appeared independent of VR (Table 2).

**Table 2:** Characteristics of meta-analyses of randomized controlled trials

Study	Population	Type of intervention	Comparison	Duration of experiment	No. of included studies	No. of participants	Outcomes	AMSTAR-2	Adverse events
Li et al., 2021 [30]	Stage 1 to 4f PD	VR-enhanced rehabilitation interventions	Usual care or exercise therapy	3 to 12 weeks	23	836	BBS + , PDQ-39 + , ADL scale +	Low	no
Elisabetta et al., 2022 [27]	PD, balance/mobility impairment but PD, preserved ability to walk independently	VR-BT training	BT	4 to 12 weeks	22	901	BBS + TUG + , Walking speed-	Moderate	Tow reported adverse events
Joseph et al., 2020 [11]	PD	VR immersion with headset to view task, nonimmersive views of task on computer or TV monitor	active (alternative therapy treatment), passive (without alternative therapy)	4 to 12 weeks	10	343	Walking speed- Stride length + , BBS + , UPDRS- PDQ-39 +	Moderate	no
Zhang et al., 2022 [29]	PD, mean age above 60 years	13 trials of commercial exergaming systems, 5 trials of exergaming systems for older adults with PD, 1 trial not mentioned	17 trials with physical training, 2 trials without exercise training	4 to 12 weeks	19	781	TUG + , 6MWT + , BBS + , FGA- Walking speed-, Stride length-	Moderate	no
Chen et al., 2020 [24]	Idiopathic PD at different disease stages	VR-based training of different setups and exercise items	Conventional balance training, No treatment Active control (strength training program) Both stationary cycling and no treatment	5 ~ 12 weeks	14	574	BBS + ABC- TUG- DGI + FGA +	Very low	One study reported effects associated with the VR training
Wang et al., 2019 [31]	PD	Basic virtual reality and augmented virtual reality	CBT Conventional gait training Muscle strength training	4 ~ 12 weeks	12	419	BBS + TUG + Stride length + Gait velocity- Walk distance-	Very low	no
Chen et al., 2020 [32]	PD	VR training VR balance training VR-based training of different platform	Physiotherapy No training Balance training CBT Traditional exercise therapy	4 ~ 12 weeks	12	360	BBS + TUG + 10MWT + MBI +	Very low	no
Wang 2021 [28]	PD	Non-immersive VR interventions	Conventional training Traditional exercise	5 ~ 12 weeks	12	525	OLS + LOS + SOT + BBS + TUG- FES + ABC + PDQ-39 +	Low	no

*PD* Parkinson’s disease, *VR* virtual reality, *BBS* Berg Balance Scale, *ABC* Activities-Specific Balance Confidence Scale, *TUG* Timed Up and Go Test, *DGI* Dynamic Gait Index, *FGA* Functional Gait Assessment, *CBT* conventional balance training, *MBI* modified Barthel Index, *OLS* The One-Leg Stance Test, *LOS* the Limits of Stability, *SOT* the Sensory Organization Test, *FES* the Falls Efficacy Scale, *PDQ-39* the 39-Item Parkinson’s disease Questionnaire, *6MWT*, 6-Minute Walk Test, *AMSTAR-2*, A Measurement Tool to Assess Systematic Reviews, *BT*, balance training, *PDQ39*, Parkinson’s Disease Questionnaire 39, *QoL*, quality of life, *UPDRS*, Unified Parkinson’s Disease Rating Scale, *10MWT*, 10-Minute Walk Test

The methodologic quality of the eight meta-analyses was assessed using AMSTAR-2. The results showed that three meta-analyses were of moderate quality [11, 27, 29], tow were of low quality [28, 30] and three were of very low quality [24, 31, 32] (Additional file 1: Table S4). Eligible meta-analysis were included according to the inclusion and exclusion criteria, notably none of the four meta-analyses reported the source of funding for the included studies (Additional file 1: Table S4).

### Outcomes and findings

Four meta-analysis included in the umbrella review was recalculated effect size, which described eight potential correlations. Pooled results from eight primal studies found VR training can large improve the basic balance ability in PD patients (TUG test; g = 0.906, 95% CI [0.195 to 1.610] BBS test; g = 0.657; 95% CI [0.365 to 0.900]), but the heterogeneity of both correlations is high (TUG:I^2^ = 84; BBS:I^2^ = 62). Stride length is also moderate improved (g = − 0.488, 95% CI [− 0.845 to − 0.130]), with low heterogeneity (I^2^ = 0). Besides, for ADL and QoL, the effect sizes were pooled from five studies and nine studies that suggested VR training can improve ADL (ADL scale; g = 0.618; 95% CI [0.319 to 0.917]) and QoL (PDQ-39; g =  − 0.277, 95%CI [− 0.505 to − 0.040]) for PD patients, with both low heterogeneity (I^2^ = 0). We found no publication bias in any of the eight outcomes by Egger's test (*P* > 0.05) (Table3).

**Table 3:** Summary of significant outcomes of virtual reality interventions for Parkinson’s disease

Study	Number of primary studies	Outcome	No. of participants	Hedges’ g (95%CI)	*P* random	I^2^	95%PI	Largest study (95%CI)	Egger's test *P* value	Grade rating	AMSTAR2 rating	Significant studies	
												Observed	*P* value
Li et al., 2021 [30]	5	ADL scale	187	0.618 (0.319 to 0.917)	5.04 × 10 − 5	0	(0.133 to 1.103)	(0.111 to 1.269)	0.579	Very low	Low	1	0.57
Li et al., 2021 [30]	9	PDQ-39	307	− 0.277 (− 0.505 to − 0.049)	1.71 × 10 − 2	0	(− 0.552 to − 0.002)	(− 1.3 to − 0.14)	0.193	Low	Low	1	0.255
Li et al., 2021 [30]	15	BBS	551	0.657 (0.365 to 0.95)	1.08 × 10 − 5	62	(− 0.359 to 1.673)	(0.218 to 1.142)	0.473	Very low	Low	7	0.476
Elisabetta et al., 2022 [27]	8	TUG	236	0.906 (0.195 to 1.617)	1.25 × 10 − 2	84.1	(− 1.556–3.368)	(2.646 to 4.636)	0.221	Very low	Moderate	4	0.86
Elisabetta et al., 2022 [27]	8	Walking speed	279	0.107 (− 0.13 to 0.344)	0.376	0	(− 0.188 to 0.402)	(− 0.717 to 0.241)	0.0997	Very low	Moderate	0	0.747
Joseph et al., 2020 [11]	3	UPDRS	75	− 0.38 (− 1.455 to 0.695)	0.488	80	(− 13.219 to 12.459)	(− 1.694 to − 0.102)	0.842	Low	Moderate	2	0.0169
Zhang et al., 2022 [29]	4	Stride length	124	− 0.488 (− 0.845 to − 0.131)	7.43 × 10 − 3	0	(− 1.273 to 0.296)	(− 1.029 to 0.161)	0.33	Moderate	Moderate	0	0.897
Zhang et al., 2022 [29]	3	FGA	107	0.37 (− 0.096 to 0.836)	0.12	26.6	(− 3.708 to 4.448)	(− 0.506 to 0.599)	0.317	Moderate	Moderate	1	0.311

*95%CI* 95% confidence interval, *AMSTAR-2* A Measurement Tool to Assess Systematic Reviews, *BBS* Berg balance scale, *FGA* Functional Gait Assessment, *MBI* Modified Barthel Index, *PDQ-39* 39-item Parkinson’s Disease Questionnaire, *TUG* Timed Up and Go test, *UPDRS* Unified Parkinson’s Disease Rating Scale, *SE-ADL* Schwab-England ADL scale, *VR-BT* virtual reality–balance training

In addition, we found three nonsignificant correlations include walking speed (g = 0.107, 95% CI [− 0.130 to 0.340]), motor function (g =  − 0.380, 95% CI [− 1.455 to 0.695]) and gait function (g = 0.370, 95% CI [− 0.096 to 0.836]); GRADE, Moderate. All significant and nonsignificant information with correlation results is presented in Table 3 and Additional file 1: Table S5, S6.

Assessment of the strength of evidence for the eight outcomes using GRADE revealed that there were two [29] moderate-quality ratings, tow low-quality rating [11, 30] (25% for two outcomes), and four very low ratings [27, 30] (50% for four outcomes). Of the five statistically significant correlations, only TUG and stride length were moderate, the rest were low or very low.

### Sensitivity analyses

We excluded RCTs with sample sizes of less than 25% of the total and reran the analysis. The results showed an elevated GRADE rating for ADL scale and walking speed (form very low to low), with no change in the remaining results (Additional file 1: Table S7). After removing the smaller RCTs, one outcome could not be meta-analyzed in the remaining studies. Using an HKSJ random effects model for sensitivity analysis of outcomes with fewer than five RCTs, the 95% CI of the yard HKSJ approach are more robust to changes estimated by the heterogeneity approach and show minimal bias under most of our simulations except for the 95% coverage condition [32]. Three outcomes with fewer than five RCTs maintained the same rank in quality after transforming the random-effects model (Additional file 1: Table S7), and the range of 95% CI was narrowed. Still, two outcomes crossed the null prior, with one moderate-quality outcome being the VR training with stride length (g =  − 0.488, 95% CI [− 0.588 to 0.388]).

## Discussion

The present umbrella review systematically assessed the efficacy of VR training for different motor performance, ADL and QOL outcomes across published meta-analyses of RCTs. The effect size (g) for each meta-analysis was recalculated with a standardized approach of random-effects analysis. The results shown that VR training can effectively improve the balance ability (TUG test; g = 0.906, 95% CI [0.195 to 1.61]; BBS test, g = 0.657, 95% CI [0.365 to 0.950], respectively), stride length (g =  − 0.488, 95% CI [− 0.845 to − 0.131]), ADL (ADL scale; g = 0.618, 95% CI [0.319 to 0.917]) and QoL (PDQ-39; g =  − 0.277, 95% CI [0.505 to 0.049]). For other motor performance outcomes, the results did not provide statistically significant evidence for an efficient impact of the VR training for walking speed (g = 0.107, 95% CI [− 0.130to 0.344]), motor function (g =  − 0.38, 95% CI [− 1.455 to 0.695]) and gait function (g = 0.370, 95% CI [− 0.096 to 0.836]). The analyzed meta-analyses showed low-to-moderate methodological quality as well as presented evidence of moderate -to-very low quality.

The findings suggest that VR training significantly improved motor performance mainly including balance ability and stride length in individuals with PD. Other review have also highlighted VR training improved motor performance in neurological patients [33]. This may be because repetitive motor exercises performed by the patients in the VR environment induced remodeling of neuronal dendrites, which led to activation of the primary sensorimotor cortex and improvement of motor abilities. [34]. In our study, we reviewed carefully the included review with meta-analysis found one study looking at stride length [29] and two assessing balance [27, 37] supported the effectiveness of VR training on motor performance. In the stride length study [29], 124 individuals with PD were randomly assigned to either the VR group (2 to 5 training sessions per week for 63 participants) or the physical training group (61 participants). In the original meta-analysis, the results showed that stride length did not show statistical significance after treatment. But on secondary analysis herein, VR was found to make a significance difference, in line with other data [38, 39]. This may have occurred because the number of RCTs included in the meta-analysis was too small and that the number of experiments had to be increased in order to reevaluate the results. For the balance focused studies, both sets of experiments showed that VR training was more significant in improving PD balance than ordinary motor interventions (such as aerobic exercise, yoga, stretching, tai chi and so on). However, no significant improvement was found in walking speed, gait function, or motor function. These results are important in the context of the scarcity of evidence-based support for VR training that can be used to generate recommendations for clinicians and guide clinical practice.

The improvement of motor function was considered the basis for improving QoL and ADL for PD patients [35, 36]. Our results also suggest that patients with PD have greater increases in quality of life after VR training. In the present meta-analysis, ADL was rated using ADL scale (MBI, UPDRS and SE-ADL), whereas the PDQ-39 commonly rated QoL. The difference of the correlations of QoL and ADL under random effects were recalculated. This demonstrated that VR-mediated improvements in both of these metrics were indeed statistically significant (*P* < 0.05). This new finding is likely the result of VR effects upon motor function, and visual, auditory, and tactile stimulation all of which could positively alter QoL and ADL. In addition, use of VR training may be more exciting than ordinary physical activity interventions. It can even be fun, providing a better immersive exercise experience to increase motivation [6, 40].

The findings in the present umbrella study are most applicable to individuals with PD undergoing motor function restoration especially for balance, stride length, ADL and QoL. It is important to note that although many experimental studies have demonstrated that VR training can improve lower extremity mobility and improve ADL, the evidence level for these trials was low. The evidence of quality in the study was rated moderate to very low for most outcomes according to the GRADE criteria. The primary reason for this was the lack of blinded participants and therapists during the study. In most situations, the intervention of each experimental participant was known to the measurer during the experiment. This would introduce observer bias and possibly confound randomization and outcome measurements. However, in some situations blinding may not be possible. In many physical therapy trials, it is rare to see two points allocated for blinded participants and therapists [41]. Another weakness with available data is that follow-up is all short-term (3 to 12 weeks). It is unclear whether the long-term use of VR training negatively impacts other physical functions. Further study can be made for increasing the length of time VR training is used in clinical practice to explore its long-term effectiveness. This will contribute to the objectivity and popularity of VR training in clinical trials and remote home applications.

In realistic PD clinical rehabilitation, VR training is not employed widely, mainly because mechanism of action of VR training in individuals with PD is unclear. and questions remain as to the duration of effect, as mentioned above [42]. On other hand, VR training still remain some limitations of real-word clinical application compared with traditional physical rehabilitation. Firstly, the cost of VR is high, and although the current study used a commercial VR, it costs more to build a specialized rehabilitation VR; secondly, there is currently no standardized VR rehabilitation protocol to guide rehabilitation physicians in clinical application, and the identification of standardized VR rehabilitation guidelines could help to promote the prevalence of VR application in clinical practice [43]. The possible adverse effects of VR training are also worth considering, such as vision loss, deafness, vertigo, and photosensitive epilepsy showed be made [44].

The present study had several limitations. First, the study focused only on the currently available meta-analyses of RCTs. Second, data was not found pertaining to VR side effects. Third, this study did not directly assess the quality of all the associated studies in each meta-analysis but focused only on the quality of the included associated studies. Fourth, limitations in source data restricted determination of VR related effects on upper extremity executive ability and cognitive function. As well, there were no data on long-term outcomes. This may be because prolonged rehabilitation interventions make it difficult for individuals with PD to adhere to established schedules. As most individuals with PD patients are elderly attendance to follow-up visits may be more difficult to obtain. Fifth, heterogeneity was high from the analysis of our study. The reason for the higher heterogeneity may be the bias in the quality of the included meta-analyses, and more high-quality studies are needed for further observation in the future.

## Conclusions

The current umbrella review identified improvements in balance, stride length, quality of life, and activity of daily living in PD patients after VR training. These results indicate that new types of VR training may be useful in rehabilitating individuals with PD. However, more long-term and large cohort clinical trials are needed to demonstrate the effects of VR training on outcomes such as psychological status and upper extremity motor performance.

### Supplementary Information

**Additional file 1: Table S1.** Search Strategy From Database Inception to August 3, 2023, for Meta-Analyses of Randomized Controlled Trials. **Table S2.** Excluded Studies with Reasons from the Search for Meta-Analyses of Randomized Controlled Trials. **Table S3.** Excluded Studies After Applying Inclusion Criteria for Overlapping Meta-analyses. Reason for exclusion of meta-analysis: either not with the largest number of primary cohort studies or the largest number of cases. **Table S4.** AMASTAR-2 assessments. **Table S5.** Summary of Significant Effects of VR Interventions Outcomes With Detail of GRADE Assessment. **Table S6.** Summary of Nonsignificant Effect of VR Interventions Outcomes With Detail of GRADE Assessment. **Table S7.** Sensitivity Analyses of Meta-analysis of RCTs.

## Data Availability

The datasets supporting the conclusions of this article are included within the article.

## Abbreviations

ABC: Activities-specific Balance Confidence Scale
ADL: Activities of Daily Living
BBS: Berg Balance Scale
CI: Confidence Interval
DL: DerSimonian and Laird
DGI: Dynamic Gait Index Test
FES: Falls Efficacy Scale
FGA: Functional Gait Assessment Test
g: Hedges' g
HKSJ: Hartung-Knapp-Sidik-Jonkam
LOS: Limits of Stability Test
MBI: Modified Barthel Index
MS: Multiple Sclerosis
OLS: One-Leg Stance Test
PD: Parkinson’s Disease
PDQ-39: 39-Item Parkinson's Disease Questionnaire
PI: Predictive Interval
QoL: Quality of Life
RCTs: Randomized Controlled Trials
SD: Standard Deviation
TUG: Timed Up and Go test
UPDRS: Unified Parkinson's Disease Rating Scale
VR: Virtual Reality
6MWT: 6-Minute Walk Test
