# Supplementary material for: Efficacy of virtual reality training on motor performance, activity of daily living, and quality of life in patients with Parkinson's disease: an umbrella review comprising meta-analyses of randomized controlled trials
Source: J Neuroeng Rehabil. 2023 Sep 30;20:133. doi: 10.1186/s12984-023-01256-y (PMC10544145; doi:10.1186/s12984-023-01256-y)
Supplement: Supplementary file 1 — Additional file 1: Table S1. Search Strategy From Database Inception to August 3, 2023, for Meta-Analyses of Randomized Controlled Trials. Table S2. Excluded Studies with Reasons from the Search for Meta-Analyses of Randomized Controlled Trials. Table S3. Excluded Studies After Applying Inclusion Criteria for Overlapping Meta-analyses. Reason for exclusion of meta-analysis: either not with the largest number of primary cohort studies or the largest number of cases. Table S4. AMASTAR-2 assessments. Table S5. Summary of Significant Effects of VR Interventions Outcomes With Detail of GRADE Assessment. Table S6. Summary of Nonsignificant Effect of VR Interventions Outcomes With Detail of GRADE Assessment. Table S7. Sensitivity Analyses of Meta-analysis of RCTs. [file 12984_2023_1256_MOESM1_ESM.docx]

**Additional Material**

Table S1 Search Strategy From Database Inception to August 3,2023,for Meta-Analyses of Randomized Controlled Trials

Table S2 Excluded Studies with Reasons from the Search for Meta-Analyses of Randomized Controlled Trials

Table S3 Excluded Studies After Applying Inclusion Criteria for Overlapping Meta-analyses

Reason for exclusion of meta-analysis: either not with the largest number of primary cohort studies or the largest number of cases.

Table S4 AMASTAR-2 assessments

Table S5 Summary of Significant Effects of VR Interventions Outcomes With Detail of GRADE Assessment

Table S6 Summary of Nonsignificant Effect of VR Interventions Outcomes With Detail of GRADE Assessment

Table S7 Sensitivity Analyses of Meta-analysis of RCTs

Table S1. Search Strategy From Database Inception to August 3,2023,for Meta-Analyses of Randomized Controlled Trials

| Database | Search term | Results |
| --- | --- | --- |
| PubMed | ("parkinson s*"[Title/Abstract] AND ("randomized controlled trials"[Title/Abstract] OR "randomized controlled studies"[Title/Abstract] OR "randomized controlled experiments"[Title/Abstract]) AND ("augmented reality"[Title/Abstract] OR "virtual reality"[Title/Abstract]) AND ("systematic review"[Title/Abstract] OR "meta-analysis"[Title/Abstract]) | 14 |
| Web of Science | ((((TS=(parkinson's*)) AND (TS=(meta-analysis)) OR TS=(systematic review) ) AND (TS=(augmented reality)) OR TS=(virtual reality) AND ((TS=(randomized controlled trials)) OR TS=(randomized controlled studies)) OR TS=(randomized controlled experiments) | 33 |
| Scoups | (TITLE-ABS-KEY("meta-analysis")OR TITLE-ABS-KEY("systematic review")) AND (TITLE-ABS-KEY("augmented reality")OR TITLE-ABS-KEY("virtual reality")) AND (TITLE-ABS-KEY("randomized controlled trials")OR TITLE-ABS-KEY("randomized controlled studies")OR TITLE-ABS-KEY(randomized controlled experiments)) AND (TITLE-ABS-KEY("Parkinson's")) | 52 |
| PsyclInfo | tiab(meta-analysis) OR tiab(systematic review)AND tiab(augmented reality) OR tiab(virtual reality)AND tiab(randomized controlled trials) OR tiab(randomized controlled studies) OR tiab(randomized controlled experiments)AND tiab(Parkinson's) OR tiab(Parkinson's*) | 5 |
| Total |  | 104 |

Table S2. Excluded Studies with Reasons from the Search for Meta-Analyses of Randomized Controlled Trials

| Reason for exclusion | References |
| --- | --- |
| Not a meta-analysis(n=41) | 1. Wu, Y. T., Wu, Y. F., & Ye, J. H. (2020). Adults with Parkinson’s disease undergoes exergaming training to improve balance: A systematic review. *International Journal of Information and Education Technology, 10*(2), 146-153. doi:10.18178/ijiet.2020.10.2.1354 2. Cano Porras, D., Siemonsma, P., Inzelberg, R., Zeilig, G., & Plotnik, M. (2018). Advantages of virtual reality in the rehabilitation of balance and gait: Systematic review. *Neurology, 90*(22), 1017-1025. doi:10.1212/WNL.0000000000005603   3.Cernich, A. N., Kurtz, S. M., Mordecai, K. L., & Ryan, P. B. (2010). Cognitive rehabilitation in traumatic brain injury. *Current Treatment Options in Neurology, 12*(5), 412-423. doi:10.1007/s11940-010-0085-6  4.Pallavicini, F., Pepe, A., & Mantovani, F. (2021). Commercial off-the-shelf video games for reducing stress and anxiety: Systematic review. *JMIR Mental Health, 8*(8). doi:10.2196/28150  5.Da Rocha, P. A., McClelland, J., & Morris, M. E. (2015). Complementary physical therapies for movement disorders in Parkinson's disease: a systematic review. *European Journal of Physical and Rehabilitation Medicine, 51*(6), 693-704.  6.Fovet, T., Jardri, R., & Linden, D. (2015). Current issues in the use of fMRI-based neurofeedback to relieve psychiatric symptoms. *Current Pharmaceutical Design, 21*(23), 3384-3394. doi:10.2174/1381612821666150619092540  7.Campo-Prieto, P., Santos-Garcia, D., Cancela-Carral, J. M., & Rodriguez-Fuentes, G. (2021). Current status of immersive virtual reality as a tool for physical and functional rehabilitation in patients with Parkinson's disease: systematic review. *Revista de Neurologia, 73*(10), 358-367. doi:10.33588/rn.7310.2021330  8.Emmanouilidis, S., Hackney, M. E., Slade, S. C., Heng, H., Jazayeri, D., & Morris, M. E. (2021). Dance Is an Accessible Physical Activity for People with Parkinson's Disease. *Parkinson's Disease, 2021*. doi:10.1155/2021/7516504  9.Thangavelu, K., Hayward, J. A., Pachana, N. A., Byrne, G. J., Mitchell, L. K., Wallis, G. M., . . . Dissanayaka, N. N. (2022). Designing Virtual Reality Assisted Psychotherapy for Anxiety in Older Adults Living with Parkinson's Disease: Integrating Literature for Scoping. *CLINICAL GERONTOLOGIST, 45*(2), 235-251. doi:10.1080/07317115.2019.1709597  10.Morales-Gómez, S., Elizagaray-García, I., Yepes-Rojas, Ó., de la Puente-Ranea, L., & Gil-Martínez, A. (2018). Effectiveness of virtual immersion programmes in patients with parkinson’s disease. A systematic review. *Revista de Neurologia, 66*(3), 69-80. doi:10.33588/rn.6603.2017459  11.Chuang, C. S., Chen, Y. W., Zeng, B. Y., Hung, C. M., Tu, Y. K., Tai, Y. C., . . . Tsai, C. H. (2022). Effects of modern technology (exergame and virtual reality) assisted rehabilitation vs conventional rehabilitation in patients with Parkinson's disease: a network meta-analysis of randomised controlled trials. *Physiotherapy (United Kingdom), 117*, 35-42. doi:10.1016/j.physio.2022.07.001  12.Hao, Z., Zhang, X., & Chen, P. (2022). Effects of Ten Different Exercise Interventions on Motor Function in Parkinson’s Disease Patients—A Network Meta-Analysis of Randomized Controlled Trials. *Brain Sciences, 12*(6). doi:10.3390/brainsci12060698  13.Lei, C., Sunzi, K., Dai, F., Liu, X., Wang, Y., Zhang, B., . . . Ju, M. (2019). Effects of virtual reality rehabilitation training on gait and balance in patients with Parkinson’s disease: A systematic review. *PLoS ONE, 14*(11). doi:10.1371/journal.pone.0224819  14.Sevcenko, K., & Lindgren, I. (2022). The effects of virtual reality training in stroke and Parkinson’s disease rehabilitation: a systematic review and a perspective on usability. *European Review of Aging and Physical Activity, 19*(1). doi:10.1186/s11556-022-00283-3  15.Feitosa, J. A., Fernandes, C. A., Casseb, R. F., & Castellano, G. (2022). Effects of virtual reality-based motor rehabilitation: A systematic review of fMRI studies. *Journal of Neural Engineering, 19*(1). doi:10.1088/1741-2552/ac456e  16.Wang, Y., Sun, X., Li, F., Li, Q., & Jin, Y. (2022). Efficacy of non-pharmacological interventions for depression in individuals with Parkinson's disease: A systematic review and network meta-analysis. *Frontiers in Aging Neuroscience, 14*. doi:10.3389/fnagi.2022.1050715  17.Pezzi, L., Di Matteo, A., Insabella, R., Mastrogiacomo, S., Baldari, C., Reiss, V. M., & Paolucci, T. (2022). How Cognitive Reserve should Influence Rehabilitation Choices using Virtual Reality in Parkinson's Disease. *PARKINSONS DISEASE, 2022*. doi:10.1155/2022/7389658  18.Marotta, N., Calafiore, D., Curci, C., Lippi, L., Ammendolia, V., Ferraro, F., . . . de Sire, A. (2022). Integrating virtual reality and exergaming in cognitive rehabilitation of patients with Parkinson disease: a systematic review of randomized controlled trials. *European Journal of Physical and Rehabilitation Medicine, 58*(6), 818-826. doi:10.23736/S1973-9087.22.07643-2  19. Wajda, D. A., Mirelman, A., Hausdorff, J. M., & Sosnoff, J. J. (2017). Intervention modalities for targeting cognitive-motor interference in individuals with neurodegenerative disease: a systematic review. *Expert Review of Neurotherapeutics, 17*(3), 251-261. doi:10.1080/14737175.2016.1227704  20. Hvingelby, V. S., Glud, A. N., Sørensen, J. C. H., Tai, Y., Andersen, A. S. M., Johnsen, E., . . . Pavese, N. (2022). Interventions to improve gait in Parkinson’s disease: a systematic review of randomized controlled trials and network meta-analysis. *Journal of Neurology, 269*(8), 4068-4079. doi:10.1007/s00415-022-11091-1  21. Özden, F. (2021). Letter to the Editor: “Comparison of virtual reality rehabilitation and conventional rehabilitation in Parkinson's disease: a randomised controlled trial”. *Physiotherapy (United Kingdom), 110*, 87. doi:10.1016/j.physio.2020.04.001  22. Khan, F., Amatya, B., Galea, M. P., Gonzenbach, R., & Kesselring, J. (2017). Neurorehabilitation: applied neuroplasticity. *Journal of Neurology, 264*(3), 603-615. doi:10.1007/s00415-016-8307-9  23. Bevilacqua, R., Maranesi, E., Riccardi, G. R., Di Donna, V., Pelliccioni, P., Luzi, R., . . . Pelliccioni, G. (2019). Non-immersive virtual reality for rehabilitation of the older people: A systematic review into efficacy and effectiveness. *Journal of Clinical Medicine, 8*(11). doi:10.3390/jcm8111882  24. García-López, H., Obrero-Gaitán, E., Castro-Sánchez, A. M., Lara-Palomo, I. C., Nieto-Escamez, F. A., & Cortés-Pérez, I. (2021). Non-immersive virtual reality to improve balance and reduce risk of falls in people diagnosed with parkinson’s disease: A systematic review. *Brain Sciences, 11*(11). doi:10.3390/brainsci11111435  25. Ernst, M., Folkerts, A. K., Gollan, R., Lieker, E., Caro-Valenzuela, J., Adams, A., . . . Kalbe, E. (2023). Physical exercise for people with Parkinson's disease: a systematic review and network meta-analysis. *Cochrane Database of Systematic Reviews*(1). doi:10.1002/14651858.CD013856.pub2  26. Sumec, R., Filip, P., Sheardova, K., & Bares, M. (2015). Psychological Benefits of Nonpharmacological Methods Aimed for Improving Balance in Parkinson's Disease: A Systematic Review. *BEHAVIOURAL NEUROLOGY, 2015*. doi:10.1155/2015/620674  27. Devos, H., Ranchet, M., Akinwuntan, A. E., & Uc, E. Y. (2015). Establishing an evidence-base framework for driving rehabilitation in Parkinson's disease: A systematic review of on-road driving studies. NEUROREHABILITATION, 37(1), 35-52. doi:10.3233/NRE-151239  28. Li, L., & Sun, Y. (2023). Research hotspots and trends of the tele-rehabilitation for stroke survivors based on CiteSpace: A review. *Medicine (United States), 102*(13), E33398. doi:10.1097/MD.0000000000033398  29. Carmignano, S. M., Fundaro, C., Bonaiuti, D., Calabro, R. S., Cassio, A., Mazzoli, D., . . . Andrenelli, E. (2022). Robot-assisted gait training in patients with Parkinson's disease: Implications for clinical practice. A systematic review. *NEUROREHABILITATION, 51*(4), 649-663. doi:10.3233/NRE-220026  30. Barry, G., Galna, B., & Rochester, L. (2014). The role of exergaming in Parkinson's disease rehabilitation: A systematic review of the evidence. *Journal of NeuroEngineering and Rehabilitation, 11*(1). doi:10.1186/1743-0003-11-33  31. Alatawi, S. F. (2021). A scoping review of the nature of physiotherapists’ role to avoid fall in people with Parkinsonism. *Neurological Sciences, 42*(9), 3733-3748. doi:10.1007/s10072-020-05015-y  32. Ong, D. S. M., Weibin, M. Z., & Vallabhajosyula, R. (2021). Serious games as rehabilitation tools in neurological conditions: A comprehensive review. *Technology and Health Care, 29*(1), 15-31. doi:10.3233/THC-202333  33. Juras, G., Brachman, A., Michalska, J., Kamieniarz, A., Pawłowski, M., Hadamus, A., . . . Słomka, K. J. (2019). Standards of virtual reality application in balance training programs in clinical practice: A systematic review. *Games for Health, 8*(2), 101-111. doi:https://doi.org/10.1089/g4h.2018.0034  34. Hampel, H., Shaw, L. M., Aisen, P., Chen, C., Lleó, A., Iwatsubo, T., . . . Vergallo, A. (2022). State-of-the-art of lumbar puncture and its place in the journey of patients with Alzheimer's disease. *Alzheimer's and Dementia, 18*(1), 159-177. doi:10.1002/alz.12372  35. Kwon, S. H., Park, J. K., & Koh, Y. H. (2023). A systematic review and meta-analysis on the effect of virtual reality-based rehabilitation for people with Parkinson’s disease. *Journal of NeuroEngineering and Rehabilitation, 20*(1). doi:10.1186/s12984-023-01219-3  36. Kashif, M., Ahmad, A., Bandpei, M. A. M., Farooq, M., Iram, H., & Fatima, R. E. (2022). Systematic review of the application of virtual reality to improve balance, gait and motor function in patients with Parkinson's disease. *Medicine (United States), 101*(31). doi:10.1097/MD.0000000000029212  37. Müller, M. L. T. M., Marusic, U., van Emde Boas, M., Weiss, D., & Bohnen, N. I. (2019). Treatment options for postural instability and gait difficulties in Parkinson’s disease. *Expert Review of Neurotherapeutics, 19*(12), 1229-1251. doi:10.1080/14737175.2019.1656067  38. Dockx, K., Bekkers, E. M. J., Van den Bergh, V., Ginis, P., Rochester, L., Hausdorff, J. M., . . . Nieuwboer, A. (2016). Virtual reality for rehabilitation in Parkinson's disease. *Cochrane Database of Systematic Reviews, 2016*(12). doi:10.1002/14651858.CD010760.pub2  39. Voinescu, A., Sui, J., & Stanton Fraser, D. (2021). Virtual reality in neurorehabilitation: An umbrella review of meta-analyses. *Journal of Clinical Medicine, 10*(7). doi:10.3390/jcm10071478  40. Amirthalingam, J., Paidi, G., Alshowaikh, K., Iroshani Jayarathna, A., Salibindla, D., Karpinska-Leydier, K., & Ergin, H. E. (2021). Virtual Reality Intervention to Help Improve Motor Function in Patients Undergoing Rehabilitation for Cerebral Palsy, Parkinson's Disease, or Stroke: A Systematic Review of Randomized Controlled Trials. *Cureus, 13*(7), e16763. doi:10.7759/cureus.16763 |
| Included non-RCTs meta-analysis(n=2) | 1. Mura, G., Carta, M. G., Sancassiani, F., Machado, S., & Prosperini, L. (2018). Active exergames to improve cognitive functioning in neurological disabilities: a systematic review and meta-analysis. *European Journal of Physical and Rehabilitation Medicine, 54*(3), 450-462. doi:10.23736/S1973-9087.17.04680-9  2. Wu, J., Zhang, H., Chen, Z., Fu, R., Yang, H., Zeng, H., & Ren, Z. (2022). Benefits of Virtual Reality Balance Training for Patients With Parkinson Disease: Systematic Review, Meta-analysis, and Meta-Regression of a Randomized Controlled Trial. *JMIR Serious Games, 10*(1). doi:10.2196/30882 |
| Not PD(n=7) | 1. Truijen, S., Abdullahi, A., Bijsterbosch, D., van Zoest, E., Conijn, M., Wang, Y. L., . . . Saeys, W. (2022). Effect of home-based virtual reality training and telerehabilitation on balance in individuals with Parkinson disease, multiple sclerosis, and stroke: a systematic review and meta-analysis. *Neurological Sciences, 43*(5), 2995-3006. doi:10.1007/s10072-021-05855-2  2. Johnson, L., Williams, G., Sherrington, C., Pilli, K., Chagpar, S., Auchettl, A., . . . Hassett, L. (2023). The effect of physical activity on health outcomes in people with moderate-to-severe traumatic brain injury: a rapid systematic review with meta-analysis. *BMC Public Health, 23*(1). doi:10.1186/s12889-022-14935-7  3. Chen, B., Liang, R. Q., Chen, R. Y., & Xu, F. Y. (2021). The effect of virtual reality training on the daily participation of patients: A meta-analysis. *Complementary Therapies in Medicine, 58*. doi:10.1016/j.ctim.2021.102676  4. Kim, Y., Hong, S., & Choi, M. (2022). Effects of Serious Games on Depression in Older Adults: Systematic Review and Meta-analysis of Randomized Controlled Trials. *Journal of Medical Internet Research, 24*(9). doi:10.2196/37753  5. Zhu, S., Sui, Y., Shen, Y., Zhu, Y., Ali, N., Guo, C., & Wang, T. (2021). Effects of virtual reality intervention on cognition and motor function in older adults with mild cognitive impairment or dementia: A systematic review and meta-analysis. *Frontiers in Aging Neuroscience, 13*, 15. doi:https://doi.org/10.3389/fnagi.2021.586999  6. Zhu, S. Z., Sui, Y. X., Shen, Y., Zhu, Y., Ali, N., Guo, C., & Wang, T. (2021). Effects of Virtual Reality Intervention on Cognition and Motor Function in Older Adults With Mild Cognitive Impairment or Dementia: A Systematic Review and Meta-Analysis. *Frontiers in Aging Neuroscience, 13*. doi:10.3389/fnagi.2021.586999  7. Cortés‐pérez, I., Zagalaz‐anula, N., Montoro‐cárdenas, D., Lomas‐vega, R., Obrero‐gaitán, E., & Osuna‐pérez, M. C. (2021). Leap motion controller video game‐based therapy for upper extremity motor recovery in patients with central nervous system diseases. A systematic review with meta‐analysis. *Sensors, 21*(6), 1-22. doi:10.3390/s21062065 |
| Not a VR training intervention(n=6) | 1. Wang, X. Q., Pi, Y. L., Chen, B. L., Wang, R., Li, X., & Chen, P. J. (2016). Cognitive motor intervention for gait and balance in Parkinson's disease: systematic review and meta-analysis. *Clinical Rehabilitation, 30*(2), 134-144. doi:10.1177/0269215515578295  2. Li, Z. L., Wang, T., Liu, H. Y., Jiang, Y., Wang, Z., & Zhuang, J. (2020). Dual-task training on gait, motor symptoms, and balance in patients with Parkinson's disease: a systematic review and meta-analysis. *Clinical Rehabilitation, 34*(11), 1355-1367. doi:10.1177/0269215520941142  3. Abou, L., Alluri, A., Fliflet, A., Du, Y., & Rice, L. A. (2021). Effectiveness of Physical Therapy Interventions in Reducing Fear of Falling Among Individuals With Neurologic Diseases: A Systematic Review and Meta-analysis. *Archives of Physical Medicine and Rehabilitation, 102*(1), 132-154. doi:10.1016/j.apmr.2020.06.025  4. Kalron, A., & Zeilig, G. (2015). Efficacy of exercise intervention programs on cognition in people suffering from multiple sclerosis, stroke and Parkinson's disease: A systematic review and meta-analysis of current evidence. *NEUROREHABILITATION, 37*(2), 273-289. doi:10.3233/NRE-151260  5. Tofani, M., Ranieri, A., Fabbrini, G., Berardi, A., Pelosin, E., Valente, D., . . . Galeoto, G. (2020). Efficacy of Occupational Therapy Interventions on Quality of Life in Patients with Parkinson's Disease: A Systematic Review and Meta-Analysis. *Movement Disorders Clinical Practice, 7*(8), 891-901. doi:10.1002/mdc3.13089  6. Perrochon, A., Borel, B., Istrate, D., Compagnat, M., & Daviet, J. C. (2019). Exercise-based games interventions at home in individuals with a neurological disease: A systematic review and meta-analysis. *Annals of Physical and Rehabilitation Medicine, 62*(5), 366-378. doi:10.1016/j.rehab.2019.04.004 |
| Not related to motor improvement(n=1) | 1. Parra, A. G., Gonzalez-Medina, G., Perez-Cabezas, V., Casuso-Holgado, M. J., Vinolo-Gil, M. J., & García-Muñoz, C. (2023). Dropout Rate of Participants in Randomized Clinical Trials That Use Virtual Reality to Train Balance and Gait in Parkinson’s Disease. A Systematic Review With Meta-analysis and Meta-regression. *Journal of Medical Systems, 47*(1). doi:10.1007/s10916-023-01930-7 |
| No data available(n=1) | 1. Lu, Y., Ge, Y., Chen, W., Xing, W., Wei, L., Zhang, C., & Yang, Y. (2022). The effectiveness of virtual reality for rehabilitation of Parkinson disease: an overview of systematic reviews with meta-analyses. *Systematic Reviews, 11*(1). doi:10.1186/s13643-022-01924-5 |

Table S3 Excluded Studies After Applying Inclusion Criteria for Overlapping Meta-analyses

Reason for exclusion of meta-analysis: either not with the largest number of primary cohort studies or the largest number of cases.

| No | Title of Article | Author (Ref) |
| --- | --- | --- |
| 1 | Effect of Virtual Reality on Balance in Individuals With Parkinson Disease: A Systematic Review and Meta-Analysis of Randomized Controlled Trials. Phys Ther. 2020;100(6):933-945. | Yi Chen 2020 |
| 2 | Effect of virtual reality on balance and gait ability in patients with Parkinson's disease: a systematic review and meta-analysis. Clin Rehabil. 2019;33(7):1130-1138 | Bo Wang 2019 |
| 3 | The Effect of Virtual Reality on the Ability to Perform Activities of Daily Living, Balance During Gait, and Motor Function in Parkinson Disease Patients: A Systematic Review and Meta-Analysis. Am J Phys Med Rehabil. 2020;99(10):917-924. | Chen Lina 2020 |
| 4 | The Effect of Virtual Reality Rehabilitation on Balance in Patients with Parkinson's Disease: A Systematic Review and Meta-Analysis[J]. Electronics, 2021, 10(9):1003- | Wenjing Wang 2021 |

Table S4 AMASTAR-2 assessments

| Items | 1 | 2 | 3 | 4 | 5 | 6 | 7 | 8 | 9 | 10 | 11 | 12 | 13 | 14 | 15 | 16 |
| --- | --- | --- | --- | --- | --- | --- | --- | --- | --- | --- | --- | --- | --- | --- | --- | --- |
| Li2021 | √ | √ | √ | √ | √ | √ | × | √ | √ | × | × | × | √ | √ | √ | √ |
| Elisabetta2022 | √ | √ | √ | √ | √ | √ | √ | √ | √ | × | √ | √ | √ | √ | √ | √ |
| Joseph2020 | √ | √ | √ | √ | √ | √ | √ | √ | √ | × | √ | √ | √ | √ | √ | √ |
| Zhang2022 | √ | √ | √ | √ | √ | √ | √ | √ | √ | × | √ | √ | √ | √ | √ | √ |
| Chen2019 | √ | × | √ | √ | √ | √ | × | √ | √ | × | √ | √ | × | √ | × | × |
| Wang2019 | √ | × | √ | √ | √ | √ | × | √ | √ | × | √ | √ | √ | √ | √ | √ |
| Chen2020 | √ | × | √ | √ | √ | √ | √ | √ | √ | × | √ | √ | √ | √ | × | × |
| Wang2021 | √ | × | √ | √ | √ | √ | √ | √ | √ | × | √ | √ | √ | √ | √ | √ |

Q1: Did the research questions and inclusion criteria for the review include the components of PICO?

Q2: Did the report of the review contain an explicit statement that the review methods were established prior to the conduct of the review and did the report justify any signifcant deviations from the protocol?

Q3: Did the review authors explain their selection of the study designs for inclusion in the review?

Q4: Did the review authors use a comprehensive literature search strategy?

Q5: Did the review authors perform study selection in duplicate?

Q6: Did the review authors perform data extraction in duplicate?

Q7: Did the review authors provide a list of excluded studies and justify the exclusions?

Q8: Did the review authors describe the included studies in adequate detail?

Q9: Did the review authors use a satisfactory technique for assessing the risk of bias (RoB) in individual studies that were included in the review?

Q10: Did the review authors report on the sources of funding for the studies included in the review?

Q11: If meta-analysis was performed, did the review authors use appropriate methods for statistical combination of results?

Q12: If meta-analysis was performed, did the review authors assess the potential impact of RoB in individual studies on the results of the meta-analysis or other evidence synthesis?

Q13: Did the review authors account for RoB in individual studies when interpreting/discussing the results of the review?

Q14: Did the review authors provide a satisfactory explanation for, and discussion of, any heterogeneity observed in the results of the review?

Q15: If they performed quantitative synthesis, did the review authors carry out an adequate investigation of publication bias (small study bias) and discuss its likely impact on the results of the review?

Q16: Did the review authors report any potential sources of confict of interest, including any funding they received for conducting the review?

**Critical domains**: Q2, Q4, Q7, Q9, Q11, Q13, and Q15.

High: No or one non-critical weakness.

Moderate: More than one non-critical weakness.

Low: One critical few with or without non-critical weaknesses.

Critically low: More than one critical few with or without non-critical weaknesses

Table S5 Summary of Significant Effects of VR Interventions Outcomes With Detail of GRADE Assessment

| Source | Outcome | Type of VR | Control | No.of studies | Sample Size  (VR/C) | Metric | Random  Effect  Size  (95%CI) | *P*  value | GRADE evidence(Not serious(NS),serious(S),very serious(VS)) | | | | | | AMSTAR-2 |
| --- | --- | --- | --- | --- | --- | --- | --- | --- | --- | --- | --- | --- | --- | --- | --- |
|  |  |  |  |  |  |  |  |  | Risk of bias | Inconsistency,I2,% | Indirectness | Imprecision | Publication bias | Overall certainty of evidence |  |
| Li et al,2021 | ADL scale | VR training | 85 | 5 | 102/85 | G | 0.618  (0.319 to 0.917) | 5.04x10^-5^ | S | NS  0 | NS | S | N/A | Very low | low |
| Li et al,2021 | PDQ-39 | VR exergames training | 146 | 9 | 161/146 | G | −0.277  (−0.505 to −0.049) | 1.71x10^-2^ | NS | NS  0 | NS | S | N/A | low | low |
| Li et al,2021 | BBS | VR training | 271 | 15 | 280/271 | G | 0.657  (0.365 to 0.95) | 1.08x10^-5^ | S | S  62 | NS | S | NS | Very low | low |
| Elisabetta et al,2022 | TUG | VR-BT | 119 | 8 | 117/119 | G | 0.906  (0.195 to 1.617) | 1.25x10^-2^ | S | VS  84.1 | NS | NS | N/A | Very low | moderate |
| Zhang et al,2022 | Stride strength | EbIs | 61 | 4 | 63/61 | G | −0.488  (−0.845 to −0.131) | 7.43x10^-3^ | S | NS  0 | NS | NS | NS | moderate | moderate |

Abbreviations: ADL: activities of daily living; EbIs: exergaming-based interventions; PDQ-39: 39-item Parkinson’s disease questionnaire; BBS: Berg balance scale; TUG: Timed Up and Go; VR-BT: virtual reality-balance training; G: Hedges’s g

Table S6 Summary of Nonsignificant Effect of VR Interventions Outcomes With Detail of GRADE Assessment

| Source | Outcome | Type of VR | Control | No.of studies | Sample Size  (VR/C) | Metric | Random  Effect  Size  (95%CI) | *P* | GRADE evidence(Not serious(NS),serious(S),very serious(VS)) | | | | | | AMSTAR-2 |
| --- | --- | --- | --- | --- | --- | --- | --- | --- | --- | --- | --- | --- | --- | --- | --- |
|  |  |  |  |  |  |  |  |  | Risk of bias | Inconsistency,I^2^,% | Indirectness | Imprecision | Publication bias | Overall certainty of evidence |  |
| Elisabetta et al,2022 | walking speed | VR-BT | 140 | 8 | 139/140 | G | 0.107  (−0.13 to 0.344) | 0.376 | VS | NS  0 | NS | NS | N/A | Low | Moderate |
| Joseph et al,2020 | UPDRS | VR training | 36 | 3 | 39/36 | G | -0.38  (−1.455 to 0.695) | 0.488 | S | VS  80 | NS | NS | N/A | Very low | Moderate |
| Zhang et al,2022 | FGA | EbIs | 52 | 3 | 55/52 | G | 0.37  (−.096 to 0.836) | 0.12 | S | NS  26.6 | NS | NS | NS | Moderate | Moderate |

Abbreviations: VR-BT : Virtual reality-balance training; FGA: Functional Gait Assessment; EbIs: exergaming-based interventions; UPDRS: Motor function assessed by unified Parkinson’s disease rating scale; G: Hedges’s g.

Table S7 Sensitivity Analyses of Meta-analysis of RCTs

| Outcome | Population | Primary analysis | | | Sensitivity analysis:  Excluding studies with small sample size(＜25%) | | | Sensitivity analysis: HKSJ method(＜5 studies) | | |
| --- | --- | --- | --- | --- | --- | --- | --- | --- | --- | --- |
|  |  | No of studies | ES (95%) | GRADE | No of studies | ES (95%CI) | GRADE | No of studies | ES(95%) | GRADE |
| ADL scale | Adult with stage 1-4 of PD | 5 | 0.618  (0.319 to 0.917) | Very low | 4 | 0.564  (0.249 to 0.878) | low | 5 | NA | NA |
| QOL | Adult with stage 1-4 of PD | 9 | −0.277  (−0.505 to −0.049) | Low | 2 | −0.539  (−0.955 to −0.124) | NA | 9 | NA | NA |
| BBS | Adult with stage 1-4 of PD | 15 | 0.657  (0.365 to 0.95) | Very low | N/A-remaining studies are not enough to conduct meta-analysis | | | 15 | NA | NA |
| TUG | PD with balance/mobility impairment and  preserved ability to walk independently. | 8 | 0.906  (0.195 to 1.617) | Very low | 3 | 1.813  (0.211 to 3.416) | Very low | 8 | NA | NA |
| Walking speed | PD with balance/mobility impairment and  preserved ability to walk independently. | 8 | 0.107  (−0.13 to 0.344) | Very low | 4 | −0.02  (−0.303 to 0.263) | Low | 8 | NA | NA |
| UPDRS | PD | 3 | −0.38  (−1.455 to 0.695) | Very low | 3 | −0.38  (−1.455 to 0.695) | Very low | 3 | -0.38  (-2.759 to 1.999) | Very low |
| Stride length | PD with a mean age of above 60 years | 4 | −0.488  (−0.845 to −0.131) | Moderate | 4 | −0.488  (−0.845 to −0.131) | Moderate | 4 | -0.488  (-0.588 to -0.388) | Moderate |
| FGA | PD with a mean age of above 60 years | 3 | 0.37  (−0.096 to 0.836) | Low | 3 | 0.37  (−0.096 to 0.836) | Low | 3 | 0.37  (-0.631 to 1.371) | Low |
